# Recent Progress in Therapeutic Treatments and Screening Strategies for the Prevention and Treatment of HPV-Associated Head and Neck Cancer

**DOI:** 10.3390/v7092860

**Published:** 2015-09-17

**Authors:** Sonia N. Whang, Maria Filippova, Penelope Duerksen-Hughes

**Affiliations:** Department of Basic Science, Loma Linda University, Loma Linda, CA 92354, USA; sowhang@llu.edu (S.N.W.); mfilippova@llu.edu (M.F.)

**Keywords:** head and neck squamous cell carcinoma, high risk HPV, HPV-related oropharyngeal squamous cell carcinoma, cancer treatments, target therapy, HNSCC, HR HPV, OPSCC

## Abstract

The rise in human papillomavirus (HPV)-associated head and neck squamous cell carcinoma (HNSCC) has elicited significant interest in the role of high-risk HPV in tumorigenesis. Because patients with HPV-positive HNSCC have better prognoses than do their HPV-negative counterparts, current therapeutic strategies for HPV^+^ HNSCC are increasingly considered to be overly aggressive, highlighting a need for customized treatment guidelines for this cohort. Additional issues include the unmet need for a reliable screening strategy for HNSCC, as well as the ongoing assessment of the efficacy of prophylactic vaccines for the prevention of HPV infections in the head and neck regions. This review also outlines a number of emerging prospects for therapeutic vaccines, as well as for targeted, molecular-based therapies for HPV-associated head and neck cancers. Overall, the future for developing novel and effective therapeutic agents for HPV-associated head and neck tumors is promising; continued progress is critical in order to meet the challenges posed by the growing epidemic.

## 1. Introduction and Background

Head and neck squamous cell carcinoma (HNSCC) is the sixth most common cancer in the world, with an incidence of over half a million new cases annually [1,2,3,4,5]. The most common tumor sites of HNSCC include the oral cavity, nasal cavity, larynx, hypopharynx, and the oropharynx [2,3,4,6,7,8]. A few decades ago, a decline in HNSCC in relation to the carcinomas of the hypopharynx and larynx was indicated [2,5,9,10,11]. This was attributed to the rise in public awareness [8,12,13,14] and the consequential decline in excessive tobacco and alcohol consumption, factors traditionally associated with this carcinoma [2,5,15,16,17]. In contrast to the encouraging trend, certain types of HNSCC have risen over the past couple of decades due to an increase in the incidence of oropharynx squamous cell carcinoma (OPSCC) [2,5,10,11], which includes cancers that form in the tonsils and at the base of the tongue [4,9,10,15,17,18,19]. This became particularly evident in patients with no history of tobacco smoking or alcohol abuse [5,16,20], arguing for the presence of an additional etiological agent [3,5,15]. The striking increase in these cancers has been attributed to the rising prevalence of human papillomavirus (HPV)-associated tumors [10,15,16,21,22,23,24].

The link between HPV and oropharyngeal carcinoma was initially suggested four decades ago, when it was still considered a risk factor [11,15]. However, it was not until the past decade that the prevalence of HPV in the head and neck has elicited considerable attention [25], and the International Agency for Research against Cancer (IARC) has now acknowledged HPV as an emergent etiological factor in the development of OPSCC [4,11,15,19,20,26]. With up to 80% of OPSCC now related to HPV [26], research reveals that the virus has undoubtedly altered the epidemiology and survival outcome landscape of head and neck carcinoma [3,21,27]. In fact, the incidence of HPV-negative head and neck squamous cell carcinomas has statistically decreased by 50%, in step with the gradual reduction of tobacco and alcohol use since the 1980s [11,17,21,25,28,29]. In contrast, HPV-positive oropharyngeal carcinomas have escalated by a dramatic 225% in the US [11,13,17,21,25,28,29], and they will represent a large fraction of the HNSCC population in the country within the next 20 years [9,21]. In fact, at the current rate of increase, OPSCC is predicted to surpass the incidence of HPV-positive cervical cancer, the archetypal HPV malignancy, in the US by the year 2020 [2,13,14,15,21,23,28,30,31,32]. Not only does this carcinoma affect the US, but it also confers a growing public health concern internationally [2,21]. Thus, the increasing epidemic of HPV-derived HNSCC is becoming a major health care issue with significant clinical ramifications [2,21].

## 2. High-Risk HPV as an Etiological Factor

HPV infection has been extensively studied in the context of its association with cervical cancer [2,9,23], the second leading cancer in women in less developed countries [15,33], and multiple studies have clearly established that HPV infection in the genitals is transmitted by sexual contact [20,34]. The factors responsible for the surge in HPV-derived HNSCC were once nebulous [29,34], but accumulating evidence now indicates that HPV-initiated OPSCC may be a result of changing sexual behaviors in the population [2,10,16,17,20,21,26,28,29,35,36]. For example, it has been demonstrated that HPV is eight times more likely to be isolated from the oral cavity of sexually experienced individuals than from the oral cavity of those who are sexually inexperienced [32,37,38,39]. Similarly, oral infection is highly correlated with multiple lifetime sexual partners, early coital debut, oral–genital sex, as well as French kissing [11,15,26,29,31,35,37,39,40]. Osazuwa *et al.*, surmised that within the US, a sexually active individual is likely to encounter an HPV infection at one or more points during their lifetime [11,41]. However, not every HPV infection develops into a carcinoma. In fact, a large majority of infections are transient and clear without any clinical manifestations [9,18,23,41,42], with 66% of infections clearing within 12 months and 90% within 24 months [9,42]. Despite the high level of clearance, the presence of high-risk HPV infection in the oral cavity has been associated with a five to fifty-fold increased risk of HNSCC development, depending on the HPV type [20,28,29,38]. Consequently, the chances of developing head and neck cancer (HNC) increase when interacting with more than 25 lifetime vaginal sex partners and/or more than five lifetime oral sex partners, according to a study conducted by D’Souza *et al.* [2,20,43,44]. Interestingly, it has been shown that an HPV infection in the head and neck is correlated with an infection in the anogenital area [10,29] as cervical cancer patients have a five-fold higher risk of head and neck cancer [32,34,45]. In addition, an increased risk for tongue and tonsil carcinomas are observed in male partners of women with cervical carcinoma [2,10,32,46], and these results have been corroborated by a match on the HPV type in those couples [29,34,47,48]. Therefore, significant accumulated evidence supports the idea that the likely transmission of this infection is primarily through oral–genital and oral–oral routes [26,34].

Since HPV-positive oropharyngeal cancers display a different etiology than do HPV-negative cancers [14,21,49], HPV-derived OPSCCs are found in a subpopulation of patients that is epidemiologically, genetically, and demographically distinct from patients presenting with the more traditional HPV-negative OPSCCs [2,9,11,22]. Unlike HPV-negative OPSCCs, which are typically found in individuals older than 60 years of age with a strong history of tobacco and alcohol consumption [11,50], HPV-related OPSCC typically appears in younger populations, between the ages of 40 and 55, with generally low levels of substance abuse [9,12,29,37,51]. This cohort of patients tends to be high functioning [28], and demonstrates a better general condition [29] as well as health [2,3,36,39,52,53,54,55]. Moreover, a recent study reported an 80% higher incidence in males than in females [2,11,19,25,32,56,57] and a lower incidence in blacks than in Caucasians (4% in blacks *vs.* 34% in their Caucasian counterparts) [2,21,32,58,59]. In addition, this patient cohort possesses higher economic status and more education [2,13]. Therefore, subjects with HPV-related HNSCC are likely to be middle-aged Caucasian males who are non-smokers and non-drinkers with a higher socioeconomic status and educational level [9,28,32].

## 3. Current Treatments and Therapies

Current therapeutic interventions for HNSCC patients include surgery, chemotherapy, and radiotherapy [6,15,52,60]. Each of these treatments have been employed at different clinics in the US [31], but currently no clinical guidelines differentiating treatment strategies between HPV-derived and tobacco-derived HNSCC exist [23,61,62]. Moreover, only a few clinical trials have made such a distinction [1,2,31,60,63,64,65,66], even though these two subsets represent separate disease entities pathologically and etiologically [24,26,31,49,57,63]. Presently, the standard therapy for head and neck cancer is determined by the tumor stage [2,4,15,64], the site of the tumor [4,15,64] and the expected functional outcomes [4], as well as by the preference of the practitioner and the patient, which include considerations of the level of organ preservation and the patient’s quality of life [2]. Head and neck cancer is classified into the following categories: early-stage or stage I/II, locally advanced or stage III/IV, and recurrent or metastatic phase [67]. Early stages of head and neck cancer are usually treated with a single-modality treatment, such as radiotherapy or surgical resection [4,12,13,15,68]. A combination of multiple therapies for superior oncologic results are required for the management of advanced stages III/IV [4,61,67]; for example, surgery with adjuvant radiation or chemoradiation with chemotherapy being added for high risk pathologic features found from the surgical specimen [2,14,35,69,70], or radiotherapy with concomitant chemotherapy [14,64,71,72,73]. Therefore, patients with advanced stages of head and neck cancer are treated through a multidisciplinary and multimodal treatment approach [50,67,68,74].

### 3.1. Surgery

Surgery is one of the standard treatments for early stage I/II HNSCC. In the past, surgical procedures sometimes consisted of extensive open transmandibular, and open pharyngotomy procedures [2,12,62,64,75] that resulted in severe morbidities including facial deformity, dysarthria, and dysphagia [15,52,53,62], especially in more locally advanced cases. Over the past 30 years, advances in radiotherapy and chemotherapy yielding favorable oncologic outcomes shifted treatment choices away from open surgery [52,55,62], until new minimally invasive trans-oral surgery (TOS) came into prominence as a viable surgical tool for early phase OPSCC [9,54,62,66,75] within the last decade, promising to reduce morbidity and mortality while improving organ preservation [9,24,53]. This new surgical approach enables resection of a tumor through the opening of the mouth without the damage to normal tissue and musculature seen in transcervical or transmandibular approaches [62,76]. Because of these advancements in technology, HPV-associated OPSCC patients may be the most appropriate subgroup to undergo a minimally invasive TOS regimen since they tend to be younger, non-smokers, and have good odds for long-term survival [9,62]. Moreover, the restoration of surgical resection as a safe treatment modality reinstituted the advantage of acquiring surgical specimens for definitive pathological staging to guide in the determination of adjuvant therapy needed. Transoral laser microsurgery (TLM) and transoral robotic surgery (TORS) are currently the principal TOS techniques utilized for head and neck carcinoma [9,28,62].

TLM is one of the procedures available for early head and neck cancer [28]. This procedure utilizes surgical apparatus already present in many medical centers, such as a laryngoscope, operating microscope, and a CO_2_ laser [28,77]. TLM is capable of conserving normal tissue by resecting the tumors *via* a direct transoral approach using transtumor cuts to assess tumor depth and microscopic magnification to aid in margin control [28,54,77]; as a result, the TLM treatment of locally advanced head and neck cancer can attain excellent cosmetic and functional outcomes [28,53].

In 2009, TORS, an alternative method for transoral surgery, became approved for small primary tumors of the head and neck region [9] and is quickly becoming a popular technique [53,54,62,75]. TORS’s magnified and angled stereoscopic visualization and articulated robotic arms aid in complex resections [2,52,53,54] as well as the performance of oncologic extirpations *en bloc* in the oral cavity [28,62,77]. In addition, TORS offers tremor filtration and high-precision motion scaling although at a significantly higher cost [28,52,54,62]. The price for a Da Vinci robotic system surpasses a million dollars, and the additional expenses for services and expendable supplies can be a limiting factor for many clinical centers [53,54,62]. However, some of the advantages of the TORS over open surgery include low rates of complications and mortality with shorter postoperative recovery time, as well as satisfactory oncological results and improved swallowing outcomes [2,9,52,53]. There is some evidence to suggest that TORS resection may allow reduced doses of adjuvant radiation with similar oncologic control and reduced treatment morbidity [53,75]. To help clarify this, the ECOG 3311 clinical trial is evaluating the de-intensification of postoperative radiation after surgical resection of HPV-associated OPSCC [61,62,76]. Therefore, these new trans-oral surgical techniques are decreasing cosmetic disfigurement while improving function and quality of life [62,78].

### 3.2. Chemotherapy

Cisplatin is the most widely used chemotherapeutic agent with the best prognostic outcome, achieving about a 90% 3-year survival rate [15,67]. Cisplatin, also known as cis-Diammineplatinum (II) dichloride or CDDP, is a DNA intercalator targeting cells that replicate at a high rate [74]. This intercalator binds to guanine residues causing crosslinks between the DNA strands, and eventually leading to cell death [74]. Studies indicate that HPV-associated patients have a higher response rate to platinum-based chemotherapy than do their HPV-negative counterparts [74]. However, the benefits of this therapy come at a price due to comorbidities such as, but not limited to, xerostomia, dysphagia, neurotoxicity and renal failure [15,52,55]. This platinum-based regimen continues to be a standard treatment for organ preservation protocols [15,72,79] as well as advanced and unresectable head and neck cancers [15,80]. Other commonly used chemotherapeutic agents consist of platinum compounds such as carboplatin; taxanes such as docetaxel and paclitaxel; methotrexate; and 5-fluorouracil [67,81,82]. These chemotherapeutic drugs are showing some promise in the treatment of HNSCC patients, however, additional agents that can target the tumor cells more specifically are under investigation. Targeted chemotherapeutic agents such as cetuximab are discussed below.

### 3.3. Radiotherapy

Historically, radiotherapy has been thought of as a conventional treatment for HNSCC and is usually a component of a multi-modal therapy plan [8,55]. Radiotherapy induces double strand breaks of the tumor cells, reducing cell viability and increasing cell cycle arrest and death [83]. Radiation treatment delivery has evolved through the decades, and advances in radiotherapy have led to the development of intensity-modulated radiotherapy (IMRT) [84,85]. IMRT delivers radiation to tumor tissues while simultaneously reducing the dosage to non-carcinogenic cells [62,86]. In this manner, IMRT can more efficiently spare healthy tissues, enhance tumor coverage, and achieve a steady dose distribution [85]. Even though IMRT has improved survival outcomes, the toxicities concomitant to irradiation continue to deteriorate a patient’s quality of life [28,86]. For instance, HNSCC treated patients have a higher likelihood of experiencing occlusive carotid artery disease and stroke [12,28]. Moreover, a considerable amount of radiotherapy-induced malignancies become apparent in HNC survivors [28]. Notwithstanding, the major cause of death in HNC survivors unrelated to cancer is cardiovascular disease associated with radiotherapy [28]. Since the HPV-dependent OPSCC population is typically younger and exhibits a favorable prognosis, the value of reducing chronic morbidities such as xerostomia [12,53], dysphagia, mucositis, lymphedema, and fibrosis is considerable [3,53,62]. Therefore, radiation protocols are actively being researched in attempts to decrease both the dosage and duration of therapy [77].

Research has shown that disease control is attainable in both HPV-related and HPV-unrelated subsets when TORS is employed as an initial surgical approach followed by chemoradiation [9,35]. Unfortunately, these patients are subject to the side effects of surgical procedures as well as those of nonsurgical interventions [9,31]. Despite the improvements in therapeutic techniques toward reducing morbidity and increasing survival, the 5-year survival rate of HNSCC patients remains at around 50% [4,6,35,57,67,75,82,87,88,89].

## 4. Management of HPV-Associated Tumors: The Debate

Clinicians are becoming increasingly aware of the need for differential therapeutic regimens between HPV-positive and HPV-negative patients [31] due to their distinct disease etiologies [14,15,22,63]. Evidence that differences in the biological aspect of these subgroups may affect their prognosis and optimal treatment is increasing [1,15,90]. For example, data collected over the past several years makes a compelling case that patients with HPV-derived OPSCC have a more favorable survival than do their matched controls, regardless of treatment strategy [1,3,20,21,22,28,31,35,37,57,60,63,65,91]. Research suggests that HPV expression corresponds with increased response rates to conventional chemotherapy [2,17,28,29,52,57,63,91], radiotherapy [1,2,16,17,22,29,57,63], and radiochemotherapy (RCT) [1,28,31,52,63,65,91,92]. Moreover, the 3-year overall survival of patients with HPV-associated OPSCC is about 75% as opposed to 50% for those with HPV-unassociated malignancies [10,24,31,37,57,63]. Additionally, studies of HPV-positive HNSCC revealed a drop of approximately 50% in recurrences, a 40% decrease in the risk of death [17,25,39] and a lower incidence of metastases than seen with their HPV-negative counterparts [2,23,37,65,93]. As impressive as these statistics look, recurrence and metastasis are still responsible for the leading cause of death in HPV-derived OPSCC [31,49,94]. In summary, patients with HPV-induced tumor report improved therapeutic responses to interventions and better survival rates due to increased sensitivity to chemotherapy and radiotherapy [1,15,20,28,31,35,95].

The reason(s) HPV-related HNSCC are associated with an improved survival outcome as compared to HPV-unrelated cancers remains speculative [9,14,60], but this difference could be ascribed to a variety of factors [17,63]. One set of explanations focuses on the patient population, indicating that the favorable prognosis of patients with HPV-associated cancers may be attributable to their younger age at diagnosis [1,2,9,17,74], their high functioning and superior performance status [2,9,17], as well as the presence of minimal tobacco and alcohol related co-morbidities [1,2,17,28,74].

An alternate or possibly complementary explanation focuses on differences in biological mechanisms. That is, even though the biologic mechanisms leading to divergent prognoses in HPV-dependent and independent oropharyngeal cancer have been elusive [14,57], the survival benefit enjoyed by HPV-associated patients could be connected to the molecular differences arising from virus-mediated activities as opposed to events that occur as a consequence of the carcinogens or mutations present in non-HPV cancer patients [43,53,63]. For example, in most tobacco-related tumors, the tumor suppressor gene *TP53* is mutated and inactive, while the *TP53* gene in HPV-infected tumors is wild-type and functionally intact, with the protein being degraded by the HPV oncoprotein E6 [2,5,30,35]. Research indicates that persistent treatment with certain therapeutic agents can suppress *E6* oncogenes, allowing the *TP53* gene to carry out its normal function [53,74]. Therefore, the presence of the wild-type *TP53* gene and the lower mutation rate [37] observed in HPV-derived SCC may enable these tumor cells to undergo an intact apoptotic response when treated with radiotherapy and/or chemotherapy, resulting in a high response rate [2,3,9,20,53].

Another possibility is that HPV-positive cancer cells express viral proteins that induce and enhance the immune response, which becomes involved in clearing cancer cells during treatment [2,8,74,96]. This theory was proposed after a cancer cell line treated with chemoradiotherapy *in vitro* demonstrated increased survival [3] and resistance to treatment [1,28] as compared to the same therapy applied *in vivo*, where the cells are surrounded by an immunologic microenvironment. Likewise, an apparent higher response in immunocompetent *vs.* immunodeficient mice further supports this finding [3]. In addition, studies indicate that the majority of HPV-infected tumor patients manifest a higher titer of T cells infiltrating the tumor [1] and a high percentage of cytotoxic CD8^+^ T cells that are specific to HPV [1,3,37] compared to non-HPV tumor patients.

Lastly, the difference in the degree of intratumor heterogeneity between HPV-dependent and HPV-independent OPSCC could contribute to their divergent prognoses. Intratumor heterogeneity refers to a tumor population comprised of subpopulations that display differing genetic makeups [28]. Assuming that certain subpopulations are more susceptible to treatment therapies than others, tumors with high intratumor heterogeneity are progressively identified as having poor therapeutic response and recurrence or metastasis [28]. HPV-driven tumors are considered to represent a homogeneous, one-agent-induced population, and are thus less intratumorally heterogeneous, possibly leading to the better therapeutic response.

To date, an effective mono-dimensional therapy approach suitable for head and neck carcinoma is not available [31]. Moreover, the classical therapies generate substantial side effects [77,96]. Traditionally, therapeutic strategies have consisted of open surgery with the option of radiochemotherapy [55,77]. The adverse effects of these therapeutic interventions have not improved in recent decades, and severe consequences associated with swallowing [15,55,77], talking [15,55,77], breathing [77], hearing [15], and even one’s countenance [15,55,77] are prevalent. The current contention lies in whether the intensity level of the therapy is too high for the cohort of HPV-positive patients that exhibit better outcomes [20,23,31,55,76]. The different therapeutic strategies all have comparable oncological effects, yet the functional complications can have a particularly long lasting effect on the rising cohort of young patients with HPV-associated head and neck cancer [2,28]. In making their decisions, clinicians are dealing with a subset of patients that will most likely reach full recovery and surpass their cancer by a few decades, and hence will be severely affected by the late sequelae of cancer treatment [2,28,52,54,93]. Consequently, an intensive multidisciplinary regimen resulting in considerable morbidity might be inappropriate for the HPV-initiated HNSCC subgroup [2,9]. Accordingly, the favorable prognosis in HPV-driven oropharyngeal cancer has prompted the progression to organ preservation strategies [23,28,55] that treat the tumor with minimal cosmetic and functional complications [19]. Therefore, evaluating the options for therapeutic de-escalation to reduce toxicity and determining treatment strategy with high efficacy to optimize quality of life is of utmost importance for this HPV-associated subpopulation [9,28,31,55,64,87,97].

Some researchers contend that concurrent radiochemotherapy may confer excess treatment [9]. Moreover, evidence has surfaced denoting the overtreatment of adjuvant chemotherapy after surgical resection in locally advanced HNSCC patients [77], accruing proponents for the de-escalation regimens. Yet the establishment of a de-intensification regimen can be challenging since nearly 10% of patients with HPV-derived tumors have a poorer prognosis and a higher likelihood of developing metastases or recurrence [9,14,31,63], demanding a more potent therapy. Some advise not to change treatment decisions or management strategy on the basis of HPV, as conclusive evidence is lacking [4,18,20,24,37,97]. Others argue that the treatment of patients with HPV-associated OPSCC should depend on the tumor phase [24], the general condition and performance status of the patient, and the expected functional outcomes [9]. Their aim is to increase the opportunities to tackle early phase carcinomas with a mono-dimensional regimen [9]. Further investigation is necessary to determine whether an alternative treatment strategy is required for HPV-associated HNC patients.

## 5. De-Intensification Trials

Clinical trials testing various de-intensification strategies for HPV-positive head and neck carcinoma patients are under examination [23,28]. The de-escalation of therapy intensity may be achieved through several different approaches [36,52]. An initial proposal was to decrease the standard dose of definitive radiotherapy or chemoradiotherapy, since radiation is considered the most toxic component of a therapeutic regimen [23,28]. An Eastern Cooperative Oncology Group (ECOG1308) phase II trial evaluated the response to chemotherapy with paclitaxel, carboplatin, and cetuximab, and based on their complete response, determined which patients could safely undergo radiation dose reduction [23,31,53,80,93]. In 2014, the investigators revealed positive initial results in patients that underwent the dose reduction [3].

Another strategy is to employ the new minimally invasive TOS technique as a primary surgical therapy [28,52]. A randomized trial, ECOG3311, evaluating whether initial transoral surgery (TORS) can allow for decreased adjuvant dose radiotherapy for patients with HPV-positive HNC is currently in progress in the US [28,37,76,93].

Another possibility is the administration of a less toxic alternate agent, such as cetuximab, an anti-epidermal growth factor receptor (EGFR) antibody [52]. The Radiation Therapy Oncology Group study (RTOG 1016) and De-ESCALaTE phase III trials are comparing conventional cisplatin concurrently with radiotherapy to the new cetuximab with concomitant radiation in HPV-driven locally advanced oropharyngeal squamous cell carcinoma (SCC) [15,23,28,31,36,37,93].

## 6. Molecular Mechanisms

Ever since the presence of HPV was demonstrated in tissues of HNSCC patients in 1983, the study of molecular mechanisms in HPV-associated HNSCC has garnered significant attention [3,15,20,98]. Insight accumulated on the molecular progression of HPV derives from the extensive research performed on cervical tumorigenesis [2,9,23,74]; consequently, cervical cancer has become the standard model for HPV studies [15,18]. With an epidemic on the horizon, it will be vital to adjust our understanding of the properties of HPV in cervical carcinoma to be applicable to head and neck carcinoma [15].

Approaches already developed for the treatment and prevention of cervical cancer may be of great help in combating HPV-derived HNSCC [15]. Nonetheless, the different anatomical and molecular aspects between cervical and oropharyngeal carcinoma must be delineated to adapt the current knowledge to the oral context [15]. For example, estrogen signaling plays a significant role in cervical cancer, while hormonal dependence is not discernible in head and neck carcinomas [15,99]. Furthermore, the cervix is not as frequently exposed to elevated amounts of cytotoxic agents and chemical carcinogens as the oropharynx [9]. The distribution of specific HPV types detected in the two cancers varies as well, revealing a broad spectrum of high-risk HPV types accounting for cervical cancer in comparison to the more limited variety observed in head and neck carcinomas [15]. Another difference observed is that, contrary to the integrated HPV form predominant in cervical cancers [100,101], the HPV genome in HNSCC samples is frequently found in both episomal and integrated forms [20,32,34,102,103,104], indicating that integration is not essential for progression of tumorigenesis in this location [15,34]. Additionally, the presence of HPV in different cancers engenders divergent prognoses [57]. That is, while HPV-driven HNSCC have better treatment outcomes, the presence of HPV in cervical cancer is associated with poor prognosis [57,105], and HPV-associated cervical cancers are considered more chemoresistant than are other gynecological tumors [106]. These differential prognoses may be due to the distinctive properties and elements characteristic of the host cancer that come into play with the virus, and might contribute substantially to the pathogenesis of HPV malignancy [57]. Despite these considerations, the molecular virology of infection is not anticipated to be significantly different in HNSCC as compared to that present in cervical cancer. The prevailing understanding of the molecular details of HPV has therefore shed light on HPV-positive head and neck cancer.

HPV is transmitted through the mucosal and non-mucosal skin epithelia [15,37]. About 200 HPV types categorized based on the HPV L1 sequence have been detected, some of which have the ability to induce carcinogenesis [15,23,33,37,107]. Nearly 40 of these HPV types affect the mucosal tissues [107] and can be stratified into low-risk (HPV 6,11) and high-risk (*i.e*., HPV-16, 18) categories, based on their ability to develop precancerous lesions and their potential to cause malignant transformation [1,15,28,33,37,108,109]. The oncogenic high-risk subtypes are expected to give rise to 5.2% [14,15,18,28,110] of cancers globally, being responsible for up to 70% of oropharyngeal [14,33], 99% of cervical [14], 88% of anal [14], and 70% of vaginal [2,14,33] lesions. Of the 20 identified carcinogenic high-risk HPV types [37,107], HPV-16 is the most rampant [25,39], accounting for more than 90% of HPV-positive oropharyngeal cancers [1,14,15,16], followed by HPV-18 [11].

The HPV is a non-enveloped, double-stranded DNA virus that displays a predilection for squamous cell epithelium [15,28,33,37,111]. The stratified squamous epithelium is composed of progenitor cells in the lower *stratum*, and as they move up the suprabasal layer [20,37], they become differentiating keratinocytes [15,74]. HPV infection occurs when small lesions or tears at the surface of the epithelium are present, granting the virus entry to the progenitor cells in the basal layer of the stratified epithelium [15,20,37,74]. Following an infection, the virus will seize the host cellular machinery to synthesize viral nucleic acids and transcribe proteins, though usually at low levels [9,15,42]. HPV then takes advantage of the differentiation process in these keratinocytes to complete its life cycle [15,42,112]. When the differentiating cells reach the top *stratum* of the epithelium, HPV will proceed with protein coat formation, assembly of the new viral components, and eventual viral release [15]. Though the process described does not normally lead to cancer, certain events can trigger HPV to transform the differentiating keratinocytes into SCC [9].

The HPV genome is composed of approximately 8,000 base pairs [109] with dual promoters that encode two separate groups of viral proteins [1,107,111,113]. The non-structural or early genes *E1*, *E2*, *E4*, *E5*, *E6*, and *E7* are involved in viral replication, and the structural or late genes *L1* and *L2* control the viral packaging [15,33,107,111]. E1 manages the replication and transcription of the virus by acting as a DNA helicase [15], and is the only viral protein with enzymatic activity [33]. E2 can regulate the HPV genome and down-regulate the expression of E6 and E7 oncoproteins by binding to their promoters [15,111]. The activity of E4 is less well understood, but findings suggest that its interactions with the intermediate filaments of the keratin cytoskeleton may assist with viral release [15,114].

The immortalizing qualities of the virus are attributable primarily to the oncoproteins E6 and E7 [1,2,15,112] with additional contributions from E5 [37]. The cooperation between these three oncoproteins and with their interacting cellular partners promotes the transformation of the host’s epithelium and maintenance of the phenotype that leads to tumorigenesis [1,15,23,37,42,112]. As currently understood, the function of E5 is to subvert immune surveillance by repressing the major histocompatibility complex (MHC) class I molecules in the host cells [42,115]. Moreover, the E5 oncoprotein, particularly E5 from HPV-16, is involved with trafficking and signaling through the EGFR pathway [42,115].

The oncoproteins E6 and E7 are constitutively expressed throughout the progression of the carcinoma [90], making them attractive targets for antiviral therapy [112,114,116,117,118,119]. In the case of cervical cancer, the elevated expression of the E6 and E7 oncoproteins is attributed to the integration of HPV into the genome of the host, in such a way as to deregulate expression of the negative regulator E2 [15,19,102,111]. However, integration seems to be less necessary for the development of HNSCC, indicating that the enhanced expression of viral oncogenes in this context can be independent of viral integration [23,103]. We can speculate that the reason for the expression of oncoproteins in episomal HPV oral cancer may be exposure to exogenously derived factors, which can synergistically work in conjunction with the virus to elicit tumorigenesis.

The central role of the oncogenic protein E6 is to inhibit apoptosis of the infected cells by accelerating the degradation of apoptotic mediators, including the well-known tumor suppressor protein p53 [109,120,121,122], thereby removing these proteins from functioning in the intrinsic apoptotic pathway [115]. The HPV E6 oncoprotein induces ubiquitination of p53 by complexing with E6AP, an E3 ubiquitin ligase [20,123]. The resulting annihilation of p53 leads to the prevention of cell cycle arrest and/or apoptosis [20,30,112,123]. E6 proteins from high risk and low risk HPV types are both able to bind to p53, however, only the high-risk types are able to carry it through to proteasomal degradation [112,124]. In addition to blocking the intrinsic apoptotic pathway through p53 degradation, E6 is able to protect host cells from extrinsic apoptosis, which is triggered by the binding of tumor necrosis factors (TNF)-family ligands to their corresponding receptors [115]. For example, E6 has been shown to bind to major players of the extrinsic apoptotic pathway such as the initiator of the caspase cascade, procaspase 8 [125,126], as well as the adaptor molecule Fas-associated Death Domain (FADD) [127,128]. E6 binding to these substrates leads to their accelerated degradation, thereby inhibiting the transmission of apoptotic signals to effector caspases such as caspases 3 and 7. As a result, E6 prevents cells from undergoing apoptosis initiated through both the intrinsic and extrinsic pathways [129].

Another oncogene, *E7*, enhances cellular proliferation by inactivating the retinoblastoma protein (pRb) and other proteins involved in the control of cell division [2,25,109,120,130,131]. The HPV E7 protein binds to the pRb-E2F complex and removes pRb from the complex, leading to the disruption of cell cycle controls [20,123,132]. Hence, a therapeutic strategy that targets these oncogenes would target the cells that have been infected and transformed by reactivating their intrinsic and extrinsic apoptotic pathways and regaining cell cycle control. Such promising avenues could potentially augment the effectiveness of current modalities while reducing toxicity and morbidities.

## 7. HPV Detection and Screening Tools

The majority of head and neck carcinomas are discovered at late stages of tumor progression, arguing for the need of a reliable detection tool that is clinically relevant to facilitate early detection of HNSCC [15,27]. Considering factors of age, stage of disease, and tobacco smoking status in these cancer patients, the most significant prognostic indicator of survival found to date is HPV status [2,3,4,8,19,20,63,64,74]. It is estimated that HPV affects approximately 70% of all carcinomas in the oropharynx and the oral cavity [2,10,21,31,34,35,39,73,85]. Moreover, since HPV-related OPSCC has a remarkably more favorable prognosis than does HPV-unrelated cancer [35], establishing HPV status through an effective screening tool will offer significant advantages.

In contrast to the case with cervical cancer, there are no reliable screening methods or routine check-ups equivalent to the Pap smear to detect early HPV neoplasia in the oral cavity [13,15,29,35]. Moreover, since the infected tissue in the oral cavity normally arises in an inaccessible location, devising and implementing such a tool for regular diagnosis becomes challenging [15,32,133], leaving it up to the patients to consistently monitor for symptoms such as continual sore throats, oral lesions, or swollen masses or glands [13,15]. Unfortunately, these relatively mild and non-alarming manifestations tend to go unnoticed quite frequently, compounding the issue that most head and neck carcinomas are identified at later tumor stages by the time of diagnosis [6,7,15,82,83]. Consequently, finding accurate and practical methods to assess the presence of HPV in the oral cavity is a high priority [2].

At this point, the technique(s) to be employed for determining the HPV status of head and neck cancers is controversial, due to variations in available methods in terms of cost, sensitivity, technicality, specificity, and reliability [2,18,20,27,28,29,134,135,136]. Three common methods of detection are currently used: Polymerase Chain Reaction (PCR), in situ hybridization (ISH), and p16 immunohistochemistry (IHC) [2,54]. In particular, the detection of the viral DNA, such as *E6* or *E7* sequences [137,138] through PCR or ISH has been a very common practice [9,139,140]. PCR is highly sensitive, detecting as little viral DNA as 0.001 copy per genome from tumor samples, plasma or salivary collections [28,141]. It can also assess the viral load [135] and identify the viral subtype by probing for the *L1* region of the HPV genome [9,28,135,138,142]. A disadvantage of focusing on the *L1* region is that this region can be compromised or deleted following integration into the host genome [138,143], thereby leading to underestimates of the presence or the viral load of HPV [22]. Furthermore, since PCR detects a region of the viral genome indiscriminately of whether it is in the integrated or episomal form, this method does not have the ability to determine the physical status of the virus nor its activity, which are essential in assessing tumor development [28,138,139]. Additionally, this method is rather expensive and is therefore only utilized in select laboratory centers [28,144]. On the other hand, ISH is highly specific in detecting viral integration status and transcriptional activity [9,28,139]. It utilizes a fluorescent-labeled probe to localize and visualize the HPV DNA in the host genome of the tumor dissection [135,138]. Diffuse signals indicate the presence of episomal HPV, while punctate signals represent the integrated forms [145]. Nevertheless, since ISH does not amplify the viral genome, this method is not as sensitive [138] or as fast as PCR. However, the procedure can be automated and has become available in certain clinical laboratories [28,135].

The detection of HPV E6/E7 mRNA is the “gold standard” validation of active HPV oncoprotein transcription, and is considered clinically applicable in the evaluation of carcinogenesis [9,27,139,146]. Since mRNA is very fragile and easily degraded, fresh or rapidly frozen samples are required for this approach [9,139]. While the detection of mRNA through reverse-transcriptase PCR or RT-PCR is technically challenging and perceived as inappropriate for routine screening [9], the novel ISH assay, RNAscope, has been met with great interest and found to be perhaps the most promising of available methods [139,146].

Another major alternative for detecting the virus is the IHC of the CDK inhibitor p16, a transcript encoded by the *CDKN2A* gene [9,54,138]. This technique has become popular due to its high sensitivity [28], technical ease, swiftness, practicality [28,37,139,144], inexpensiveness [28,37,139,144], and adequate consistency with PCR and ISH [28]. p16 is considered a suitable surrogate marker of HPV infection [9,20], and is biologically relevant because its overexpression corresponds closely to the transformation of infected cells [15,138]. p16 becomes up-regulated when E2F is released from the E2F-pRb complex after pRb is degraded by E7 [9,15,20,37,96]. This method of detection is the most widespread across multiple clinical centers [37,139]. It should, however, be noted that not all tumors that test positive for p16 contain HPV [37]. Across various tests, HPV infection has not been identified in approximately 10%–20% of p16^+^ head and neck carcinomas [37,139]. Since the practice, interpretation, and reporting of p16 IHC differ, in some cases its prognostic diagnosis can be misinformative and hence unreliable as a stand-alone method [2,9,28,139].

Many investigators propose that using RT-PCR to detect the presence of E6/E7 mRNA may be suitable as a gold standard for fresh samples, since the expression of these two oncogenes is characteristic of a functional HPV infection and cell transformation [9,17,19,27]. However, this method requires further examination [139]. According to one study, the employment of HPV-PCR or p16 IHC alone is not very reliable or clinically adequate [147]; notwithstanding, Dalianis *et al.* reported that a HPV DNA test such as PCR in addition to an evaluation of p16 overexpression through IHC is regarded as “specific and sensitive as utilizing a gold standard” [9,17,19,145,148]. Yet another panel of experts has suggested a “cost-efficient” stepwise algorithm to reliably determine HPV infections, which includes an initial testing of p16 through IHC followed by an HPV ISH to confirm the IHC results [28,139]. If the tests provide conflicting results, a PCR or an ISH probe for specific HPV types can be utilized [28]. This sequence of methods is thought to provide the highest specificity for determining HPV status [20,139]. Others have suggested variations of these detection methods and proposed a variety of combinations [9,136,139]. In order to standardize the detection methods in clinical settings and to design reliable clinical research, a unanimous agreement on the most reliable detection tool(s) for HPV status is required and requisite [27,28].

## 8. Prophylactic Vaccines

A steep upward shift in the incidence of HPV-derived HNSCC demands a search for a vaccine that can avert the infection of oral HPV before an opportunity to develop a malignant lesion arises, especially considering the lack of a reliable routine screening tool for those at risk of oropharyngeal SCC [15,21]. Past vaccines have been effective at immunizing against viruses such as influenza and varicella, and such prototypes should help in the development of prophylactics against oral HPV infection [15].

Preventive vaccines against HPV in the cervix have been developed and have become available to the public within the past decade [9]. The first prophylactic vaccine to be approved was Gardasil, a quadrivalent vaccine that prevents infection from high-risk HPV types 16 and 18 as well as the low-risk HPV types 6 and 11 [15,42]. Cervarix has been developed as a bivalent vaccine that immunizes against HPV types 16 and 18 [15,42]. Both prophylaxes encompass the predominant high-risk HPV types that are found in cervical malignancy, whereas the quadrivalent vaccine also targets genital warts and contains in addition the two most prevalent non-oncogenic viral types [15,28]. Despite the fact that Cervarix excludes the low-risk HPV types, a study that compares both prophylaxes indicated that Cervarix is able to produce a stronger antibody response than Gardasil against the two oncogenic HPV types [42]. Phase III trials of these vaccines established efficacy and safety in the protection against anogenital HPV infections, lesions, and warts, but these prophylaxes have not been certified for the immunization of HPV infection in the head and neck region [9,15]. Notwithstanding, there is great potential that the current HPV vaccination will prevent oral HPV infection [9,19]. A trial that was originally intended to examine the efficacy of the HPV vaccine in cervical infections has collected oral rinses that showed encouraging results of the vaccine’s effectiveness in obviating HPV infection from the oral cavity [11,13,32,133].

In contrast to the large diversity of high-risk HPV types observed in cervical carcinoma [15], HPV types 16 and 18 constitute over 95% of HPV-positive tonsillar and oropharyngeal cancers [11,19,35]. Hence, the current prophylactic vaccines can be highly effective at preventing HPV-derived HNSCC, since they encompass the primary HPV types that are causal of OPSCC [15]. Moreover, although clinical evidence supporting their efficacy in the prevention of head and neck cancers is not yet documented [9,35], these vaccines have demonstrated that they can induce a systemic robust humoral response against the oncogenic HPV types 16 and 18, and hence should in principle be efficacious against oral infections [9,15,20]. Ongoing clinical trials are currently assessing the effectiveness of the quadrivalent HPV vaccine against HPV infection in the oral cavity [34]. The effect of these prophylactic HPV vaccines on oropharyngeal HPV infection and HPV-derived head and neck cancer will be clearer once further results are obtained [32,35,42].

## 9. Therapeutic Vaccines

Therapeutic vaccines for HPV-driven malignancies are currently undergoing clinical investigations [20,23]. Unlike the previously described prophylactic vaccines, which offer no protection against individuals already infected with HPV [2,35,112,116,120], therapeutic vaccines are intended to treat the individual by eliciting a cell-mediated response that can recognize and attack an established dysplasia or persistent infection [23,34,107]. Moreover, in contrast to prophylactic vaccines, which incite an antibody-mediated humoral response to clear the virus and to prevent access to the squamous epithelium, therapeutic vaccines must activate the T cell-mediated immune system to destroy the existing HPV-infected cells and prevent them from developing into carcinomas [42,111,118]. This can be challenging for immunocompromised patients because of their weakened immune system; hence, these vaccines are anticipated to be most effective in immunocompetent individuals.

In the design and development of therapeutic vaccines, HPV-16 E6 and E7 oncoproteins have become popular viral targets since they are consistently expressed in HPV malignancies and are critical for transformation [23,90,107,116,117,118,119]. Moreover, in contrast to tumorigenic antigens derived from mutated or overexpressed self-proteins, viral E6 and E7 are entirely foreign proteins, which express numerous antigenic epitopes and thus contribute toward an enhanced immune response [23,116,119]. More importantly, only the infected cells will express these viral proteins, making them ideal targets for therapy of HPV-derived cancers [23,118]. A majority of clinical trials for therapeutic vaccines are in their early phase and have focused on feasibility, immunogenicity, and safety [20,114]. Multiple vaccines are currently being explored as potential therapeutic strategies including DNA vaccines, peptide and protein vaccines, cell-based vaccines, as well as bacterial and viral live vector vaccines [20,23,107,116,117,118].

Due to their safety, ease of production, purity and stability, DNA vaccines have become attractive therapeutic candidates for HPV-associated HNSCC [23,107,111,116,118,119]. DNA vaccines introduce plasmid DNA into the host and promote its transcription and immune presentation of the encoded HPV proteins by the transfected cells [107,118,119]. This MHC presentation elicits T cell-mediated and/or antibody-mediated responses that attack the encoded antigen [107,118,119]. However, DNA vaccines can have low immunogenicity because they lack the ability to spread the DNA from the transfected cells and amplify it in the neighboring cells [111,119]. Despite such limitations, significant results from the therapeutic HPV DNA vaccine studies have progressed to various clinical investigations [119]. For example, a phase I trial at Johns Hopkins University is evaluating a DNA vaccine targeting HPV-16 E7 antigens in patients with advanced HPV-16-positive OPSCC [23,119,149]. This vaccine encodes for HPV-16 E7 fused to the immuno-modulatory agent calreticulin, a protein that can stimulate natural killer T cells and enhance MHC class I antigen presentation [23,117,119,149].

In contrast, peptide vaccines are taken up by antigen presenting cells (APC) directly without the need for encoding and are loaded onto MHC molecules for antigenic presentation [23,107]. This leads to activation of an antigen specific T cell response and putative elimination of infected cells [107]. Peptide vaccines are safe, stable, and easily prepared, but have poor immunogenicity [107,111,119]. Some adjuvants used to circumvent the low immunogenicity include costimulatory molecules, cytokines, chemokines, and Toll-like receptor (TLR) ligands [111,119]. Specific examples include calreticulin, Montanide ISA-51, and GM-CSF, [2,23,111,117]. Another disadvantage with respect to peptide vaccines is that they are MHC restricted, which limits their widespread use [111,119]. However, this restriction can be overcome by the use of overlapping long peptides that harbor several epitopes of the antigen [111]. One study has devised an HPV peptide vaccine composed of synthetic long overlapping peptides that encompass the E6 and E7 oncoproteins of HPV type 16 [42,90,111]. Additionally, a phase II clinical trial of this peptide vaccine with the adjuvant Montanide ISA-51 resulted in the mounting of a complete vaccine-induced immunologic response [42,90,111].

Protein vaccines are similar to peptide vaccines in many ways, but they can bypass MHC restriction since the protein contains a variety of antigenic epitopes [111,118]. Additionally, protein vaccines are loaded onto MHC class II molecules, creating primarily a humoral response instead of a cell-mediated response [111,118]. A phase II trial of the HspE7 protein-based vaccine, which is a chimeric protein composed of HPV-16 E7 and a Bacille Calmette-Guerin (BCG) heat shock protein (Hsp65), yielded modest results [107,118]. TA-CIN, a fusion protein composed of HPV-16 E6, E7, and L2, represents advancement in the field of HPV vaccination because it combines therapeutic as well as prophylactic vaccines. This protein-based vaccine has progressed to clinical trial [90,111].

The cell-based vaccine technique entails the pulsing of dendritic cells (DC) with an antigen [107,119], allowing for the presentation of epitopes, such as those derived from HPV E7, in association with MHC molecules, and is capable of eliciting a high immunologic response [107,111]. A phase I study has shown the approach to be safe and immunogenic, and a phase II trial is underway [107]. However, the production of this vaccine is lengthy, taxing, and expensive [111,119] due to the need to isolate immature dendritic cells from the patient, transfect or pulse the autologous DCs with the specific antigen, allow the DCs to mature, and expand the DCs *ex vivo* before injecting them back into the patient [111,118].

A live vector, consisting of either a bacteria or a virus, can be employed to deliver antigens such as those found in the E6 and E7 oncoproteins to the host APCs in order to enhance antigen presentation and the induction of a cell-mediated response [107,111,118]. These vectors generate a strong immune response by facilitating the spread and expansion of oncoproteins [107,111,118]. However, the disadvantage is that these live vectors could incite an immune response against the vector itself since it is intrinsically pathogenic and foreign to the host [107]. A bacterial vector-based vaccine composed of a genetically modified strain of *Listeria monocytogenes* fused to E7 has shown the ability to cause regression of solid tumors and has progressed to phase I clinical studies in oropharyngeal cancer patients [107,111,118,149]. Another group designed a vector vaccine using an integrase defective lentiviral vector (IDLV) to deliver a HPV-16 E7 protein fused to calreticulin [2,111,117]. A preclinical study revealed that a single intramuscular injection eradicated 90% of early stage tumors [2,117]. These encouraging outcomes along with emerging therapeutic vaccine trials may imply that an immunotherapeutic vaccine for immunocompetent patients shows a promising future [2,117].

## 10. Targeted Therapies Directed against Growth Factor Receptors

Current treatment for HNSCC patients is confined to standard therapies, such as irradiation, surgery, and chemotherapy [60,67]; and despite continued advances in these classic clinical modalities, survival rates remain comparable and many patients experience long-term side effects [15,60,82,150]. Consequently, advancements in molecular research have made the identification of targeted therapies an attractive therapeutic approach due to its purported reduced toxicity and improved efficacy [15,150].

We have come a long way in understanding the molecular biology of head and neck cancer over the past few decades [68]. Interestingly, the EGFR has been shown to be frequently elevated in over 90% of HNSCC patients [2,4,67,71,88,150]. EGFR contributes to the pathogenesis of HNSCC such that its overexpression is closely related to low survival, distant metastases, and radioresistance [4,36,67,71,88,150]. Studies have indicated that low EGFR levels in HPV-positive tumors were correlated with favorable therapeutic outcomes, while high EGFR levels were associated with poor survival [34,60,88,150,151].

The role of EGFR is to transmit signals to intracellular pathways that regulate a host of cellular activities including proliferation, cell cycle progression, apoptosis, migration, metastasis, differentiation and angiogenesis [36,60,80,151]. Among the mechanisms attributed to overexpression of EGFR are deregulation of *TP53* and amplification of *EGFR* [67]. Thus, this extracellular domain has been an attractive and prominent therapeutic target for treatment intervention [60]. Several agents directed against EGFR have been produced, of which monoclonal antibodies (mAb) and small tyrosine kinase inhibitors (TKIs) have been shown to be the most effective [68,150]. The mAbs bind to the extracellular binding domain of this receptor, while TKI’s bind to the cytoplasmic side of EGFR and influence downstream molecular pathways [2,57,68,80].

Cetuximab is a recombinant chimeric immunoglobulin (Ig)G mAb, specifically targeting the extracellular domain of EGFR [2,60,80,150]. This mAb has been the most extensively studied of the anti-EGFR antibodies [150] and is the first and only targeted therapy approved for head and neck carcinoma [14,28,68,71,80]. Food and Drug Administration (FDA) approval of cetuximab (Erbitux, Merck; Darmstadt, Germany) was established in 2006 after a phase III randomized study yielded remarkable results in the overall survival of HNSCC patients when cetuximab was used in conjunction with radiotherapy (a survival of 45.6% *vs.* 36.4% for radiotherapy alone) [4,15,28,60,67,80,152]. Therefore, cetuximab is recommended for the treatment of locally advanced HNSCC in combination with radiation and in recurrent/metastatic disease either as a monotherapy or in conjunction with platinum-based chemotherapy and 5-fluorouracil [15,23,67,71,80,150]. Several clinical trials are active including the Radiation Therapy Oncology Group (RTOG1016) trial, which compares cetuximab to cisplatin along with radiation in locally advanced disease [15,23,28,36,37,80,93]. This study will determine whether the less toxic cetuximab can replace cisplatin as part of a de-intensification protocol in HPV-derived HNSCC [37,80].

Other fully humanized IgG anti-EGFR antibodies under consideration include zalutumumab (HuMax-EGFr, Genmab, Copenhagen, Denmark) and panitumumab (Vectibix, Amgen; Thousand Oaks, CA, USA), and these are being investigated in phase II and III studies [2,23,37,67,80]. A phase II trial on nimotuzumab (YM Biosciences; Ontario, Canada), a recombinant humanized mAb, has demonstrated remarkable outcomes [67,68]. These antibodies could potentially be used as substitutes for cetuximab [2].

EGFR TKIs have also demonstrated some clinical activity in HNSCC but without as much success as seen with the mAbs [57,80]. The small molecule TKIs gefitinib and erlotinib showed no efficacy in recurring and metastasizing tumors [68,80]. A phase II trial of gefitinib on recurrent or metastatic head and neck cancer produced a low response rate [80], and ECOG-E1302, a phase III randomized study, evaluated gefitinib in addition to docetaxel in recurrent or metastatic head and neck cancer but was terminated before its completion [80]. Despite these disappointments, other EGFR targets have yielded some early encouraging results [24]. Lapatinib, a dual reversible tyrosine kinase inhibitor of EGFR/HER2, is in a phase III trial assessing its efficacy in the maintenance of treatment [60,71]. Afatinib, also known as BIBW2992, is an irreversible dual tyrosine kinase inhibitor of EGFR/HER2 [71,80]. A randomized phase II trial is comparing cetuximab to afatinib in patients with recurrent or metastatic HNSCC where cisplatin has been unsuccessful [71,80].

EGFR is involved in downstream intracellular pathways such as the PI3K/Akt/mTOR pathway. Alterations in the phosphoinositide 3-kinase (PI3K) pathway have been found in patients with head and neck cancers, and appear even more predominately in patients with HPV-derived tumors [24,57,153]. These alterations may contribute to tumor resistance to anti-EGFR therapy [24]. Hence, targeting PI3K is a reasonable strategy for OPSCC treatment, and trials in phases I and II are in progress [24]. Research on the mammalian target of rapamycin (mTOR) inhibitors rapamycin, everolimus, and temsirolimus have shown mTOR suppression and delayed tumor advancement [87,150,154]. Additionally, rapamycin has been revealed to synergize with platinum-based chemotherapy in the eradication of OPSCC [87]. There are numerous trials in progress of mTOR inhibitors concomitant with different therapeutic modalities for head and neck carcinoma [87].

Vascular endothelial growth factor (VEGF) is another type of growth factor and is considered one of the most critical angiogenic cytokines in tumor vasculogenesis [83,150]. Target agents have been developed to block its receptor, VEGFR. Bevacizumab is a monoclonal antibody against VEGFR that is being explored in conjunction to other anti-EGFR therapies [83,150,155]. Sorafenib and sunitinib are tyrosine kinase inhibitors directed against VEGFR that have revealed notable therapeutic results in different human cancer cells with tolerable toxicity, and are showing encouraging results in OPSCC [154,156].

## 11. Targeted Therapies Directed against HPV Oncoproteins

**Figure 1:**
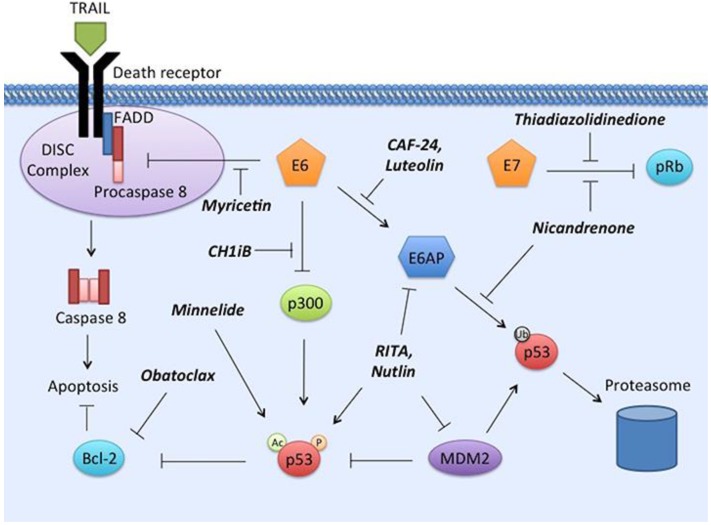
Involvement of small molecule inhibitors on cellular pathways affected by the E6 and E7 HPV oncoproteins.

Determining the molecular differences between HPV-dependent and HPV-independent head and neck cancers will be crucial in the discovery of therapeutic targets specific for HPV-dependent malignancies [15]. Various investigations have indicated that the HPV oncogenes E6 and E7 or their substrates may be efficacious anti-cancer targets [31,157]. However, approaches targeting the oncogenes have only reached very early phases of development, in contrast to the late-phase developments attained by agents targeting growth factor receptors [158]. Therapeutic agents targeting the viral oncoproteins include synthetic peptides [159], RNA aptamers [33,109], ribozymes [33,159], transcription factors [160], intrabodies [160], anti-sense oligonucleotides [33,160], small interfering RNA (siRNA) [30,33,159], and small molecule inhibitors [159]. Because small molecule inhibitors can be easily delivered and absorbed by tumor cells [159] and since they are flexible for medical use [161], they have gradually surfaced as a treatment option with notable efficacy and low toxicity (Figure 1) [30].

The interaction between E6 and E6AP represents an attractive antiviral target, as agents that target this interaction may be able to inhibit the degradation of p53 and sensitize cells to agents that induce apoptosis [30,33,90,112]. One study has identified small molecules that bind to the oncoprotein E6 with great affinity [120]. In this study, the novel flavone CAF-24 and the naturally occurring flavonoid luteolin were shown to inhibit the E6-E6AP interaction by binding to the hydrophobic site between these two proteins [120]. This strategy inhibits the oncoproteins from binding to their cellular partners, thus inhibiting their oncogenic activities [120,160]. Preventing the binding of E6AP and thus the degradation of p53 can reactivate the apoptotic pathways, enhancing the outcome of available therapies [30,120,162].

A small molecule that has been widely studied in multiple types of cancer is the p53 protector, RITA (Reactivation of p53 and Induction of Tumor cell Apoptosis) [159,163]. This molecule targets p53 by changing its conformation and protecting it from binding to molecules such as E6AP and E6 that facilitate ubiquitination [33,112,159]. In this way, p53 is rescued and the apoptotic pathway reactivated, leading to the loss of tumor cells [159].

A similar approach is taken by the non-peptide small molecule compound Nutlin-3A, an imidazoline analog and potent MDM2 antagonist. Nutlin causes substantial cell death in a variety of wild-type p53 expressing cell lines [30]; however, its activity appears to be moderate as compared to RITA [159]. Another promising molecule that reactivates the wild-type p53 is Minnelide, a triptolide analog, which has shown to induce apoptosis in HPV-positive HNSCC tumors *in vitro* as well as *in vivo* [30]. CH1iB is a novel small molecule that also reactivates p53 function by inhibiting E6 from binding to p300 and thereby allowing p300 to acetylate p53 [164]. This acetylation increases p53 stability and transcriptional activity, prompting the active p53 tumor suppressor pathway to induce apoptosis when cells are treated with chemotherapeutic agents [164]. A preclinical study of Obatoclax, a small molecule antagonist of the Bcl-2 family (B-cell lymphoma 2 (Bcl-2) is a downstream substrate of E6 that is associated with resistance to treatment [151]), indicates some therapeutic value in the treatment of oropharyngeal carcinoma [165].

Another attractive target is the interaction of E6 and caspase 8, a protein involved in the extrinsic apoptotic pathway [166]. The extrinsic apoptotic pathway can be activated by several TNF-family ligands including TNF-related apoptosis-inducing ligand (TRAIL). TRAIL can initiate apoptosis in tumor cells with expression of TRAIL-specific receptors, namely DR4 and DR5 [167], and TRAIL-therapy is considered a promising anti-tumor approach. Binding of ligands to the receptor activates the apoptotic cascade, which starts with the formation of the death-inducing signaling complex (DISC) composed, in many instances, of the receptor, FADD and the initiator caspase, procaspase 8. The assemblage of this complex results in cleavage and activation of procaspase 8. E6 interferes with this process by binding to procaspase 8 and FADD, accelerating their degradation and preventing the successful completion of the apoptotic cascade [125,126,127,128]. If therapeutic agents such as small molecules could inhibit E6 from binding to procaspase 8 and FADD, it would restore the normal functioning of the apoptosis pathway. Proof of principle for this approach was demonstrated by the flavonol myricetin, which was able to prevent the binding of E6 to caspase 8, showing potential for reactivating the extrinsic apoptotic pathway [166]. Further studies on the identification, optimization and evaluation of small molecules of E6 inhibitors are currently underway.

Another strategy is to inhibit the interaction between E7 and pRb, thereby preventing E7 from inhibiting pRb’s ability to inhibit cell division. The small compound thiadiazolidinedione inhibits HPV-E7 from disrupting the pRb-E2F complex by blocking the E7-pRb interaction [168]. Lastly, a small compound, namely nicandrenone, has demonstrated the ability to target the sites of both the E6-p53 and E7-pRb1 interactions, thereby blocking the transformative activities of both viral oncoproteins [169]. All the above strategies can lead to the development of efficient therapies against HPV-driven OPSCC and could be used in combination with current therapies to induce tumor cell death and reduce the undesirable side effects of current treatments.

Research into small molecules useful for the treatment of HPV-dependent cancers is ongoing and encouraging. However, concepts developed during studies conducted on cervical cancer will have to be assimilated and translated to oropharyngeal carcinoma. Further developments in our understanding of the molecular biology underlying the development of HNSCC will be necessary to refine the efficacy of these early phase agents.

## 12. Conclusions and Future Directions

The current epidemic of HNSCC has sparked significant interest in the role of HPV in oncogenesis, and the emergence of HPV-positive head and neck cancer has shifted the demographic of HNSCC from an older population to a younger generation. Current treatments, which consist of transoral surgery, platinum-based chemotherapy, and intensity-modulated radiotherapy, are increasingly recognized as requiring improvements. While advances in standard therapies have improved outcomes, the new group of younger patients is at high risk of morbidity and consequently a compromised quality of life. Therefore, the demand for major progress in the therapy and diagnosis of HPV-associated carcinoma remains current and compelling [35]. The better prognosis of HPV-related OPSCC has broached topics of de-escalation strategies [77], leading to the emergence of various de-intensification trials for HNSCC. With this concern in mind, standardizing a screening method for HPV status would help in diagnosing and delivering appropriate treatments to this subpopulation. The commercially available HPV prophylactic vaccines have had a profound effect in the prevention of HPV infection in the context of cervical cancer, but their efficacy has not yet been proven in the context of HPV-dependent head and neck carcinomas. Ongoing trials are anticipated to address this issue. A preventive vaccine would mitigate the epidemic long-term, but will not address the more urgent issue of treating patients with existing HPV infections. Hence, the development of therapeutic vaccines has the potential to meet a pressing need for better treatments of HPV-associated tumors in immunocompetent OPSCC patients. Additionally, targeted therapies of growth factors potentially have a more widespread use, and they have progressed in clinical trials, though with mixed results and varying success.

Several advances in biotherapy have led to the identification of a number of small molecular compounds with the potential for contributing to the development of less toxic treatments. The field of small molecular targeted therapy is in its infancy, but current findings are encouraging, advocating for the rapid progression of the field. The studies presented above reveal the urgency of the burden and the impetus to identify better targets and antiviral therapies effective in attenuating the incidence of HPV infection and counteracting the growing epidemic of HPV-associated head and neck cancers [112].
